# Computational evaluation of laparoscopic sleeve gastrectomy

**DOI:** 10.1007/s13304-021-01046-y

**Published:** 2021-04-04

**Authors:** Ilaria Toniolo, Chiara Giulia Fontanella, Michel Gagner, Cesare Stefanini, Mirto Foletto, Emanuele Luigi Carniel

**Affiliations:** 1 Department of Industrial Engineering, University of Padova, Via Venezia, 1, Padova, Italy; 2 Centre for Mechanics of Biological Materials, University of Padova, Padova, Italy; 3 Department of Surgery, Hôpital du Sacré-Coeur de Montréal, Montreal, Canada; 4 The BioRobotics Institute, Sant’Anna School of Advanced Studies, Pontedera, Italy; 5 Healthcare Engineering Innovation Center (HEIC), Khalifa University, PO Box 127788, Abu Dhabi, UAE; 6 Bariatric Unit, Week Surgery, Padova University Hospital, University of Padova, Padova, Italy

**Keywords:** Bariatric surgery, Laparoscopic sleeve gastrectomy, Bioengineering, Computational modeling

## Abstract

LSG is one of the most performed bariatric procedures worldwide. It is a safe and effective operation with a low complication rate. Unsatisfactory weight loss/regain may occur, suggesting that the operation design could be improved. A bioengineering approach might significantly help in avoiding the most common complications. Computational models of the sleeved stomach after LSG were developed according to bougie size (range 27–54 Fr). The endoluminal pressure and the basal volume were computed at different intragastric pressures. At an inner pressure of 22.5 mmHg, the basal volume of the 54 Fr configuration was approximately 6 times greater than that of the 27 Fr configuration (57.92 ml vs 9.70 ml). Moreover, the elongation distribution of the gastric wall was assessed to quantify the effect on mechanoreceptors impacting satiety by differencing regions and layers. An increasing trend in elongation strain with increasing bougie size was observed in all cases. The most stressed region and layer were the antrum (approximately 25% higher stress than that in the corpus at 37.5 mmHg) and mucosa layer (approximately 7% higher stress than that in the muscularis layer at 22.5 mmHg), respectively. In addition, the pressure–volume behaviors were reported. Computational models and bioengineering methods can help to quantitatively identify some critical aspects of the “design” of bariatric operations to plan interventions, and predict and increase the success rate. Moreover, computational tools can support the development of innovative bariatric procedures, potentially skipping invasive approaches.

## Introduction

Obesity is currently considered a global epidemic, and its prevalence has steadily increased since the early 1980s [1]. It is estimated that 1.9 billion adults are overweight, and approximately 604 million are obese; these numbers are expected to increase in the future. The prevalence of obesity is higher in women than in men [2] and dramatically increasing among the younger population [3].

Obesity is associated with multiple comorbidities and a high rate of fatalities, reaching 3.4 million in 2010 [4], with a strong impact on the healthcare system. In the US, where two-thirds of people are obese or overweight, the obesity-related medical costs amount to $209.7 billion, more than 20% of the total annual healthcare spending [5].

Due to the high efficacy, success rates and spreading of laparoscopic approaches, bariatric surgery (BS) is considered the best treatment for people affected by severe obesity [6]. Encouraging results have been recorded in terms of improvement or remission of type 2 diabetes, hypertension, dyslipidemia, and multiple other comorbidities [6]. According to the International Federation for the Surgery of Obesity and Metabolic Disorders (IFSO) global registry, the number of surgical operations increased significantly between 2014 and 2018, reaching 394,431 operations performed in 51 countries in 2018 [7]. Laparoscopic sleeve gastrectomy (LSG) is one of the most performed bariatric operations, followed by Roux-en-Y gastric bypass (LRYGB), one anastomosis gastric bypass (OAGB) and gastric banding (GB) [7].

LSG is a well-established primary bariatric procedure [8] that is considered easy to perform and allows for early discharge [9]. LSG can achieve stable and significant weight loss (approximately 82% of patients lose more than 50% of their excessive weight), improve comorbidities [10] and is considered safe (0.03% mortality rate) [9], although it is still affected by early and late complications and side effects. The most controversial issue for LSG is gastroesophageal reflux disease (GERD) due to both an increase in the esophagogastric junction angle and a significant reduction of the stomach. In fact, the angle of the gastroesophageal junction tends to increase from approximately 35° to 51° after LSG, while the gastric capacity is reduced by more than 80%; these changes seemingly correlate with reflux events [11]. It is important to address GERD because this condition can hamper LSG results and can force conversion to RYGB [12]. However, preserving antrum and LES anatomy seems not induce “de novo” GERD [13]. Most surgeons use 32–38 Fr bougies, and the choice is mainly based on personal preference rather than patient characteristics [14, 15], indicating that a more customized approach is needed.

From in vivo measurements reported by Yehoshua et al. [16], the mean volume of the residual stomach is approximately 129 ml (90–220 ml) after LSG with a 50 Fr orogastric tube. However, the residual volume of a sleeved stomach seems to increase with time, and a significant inverse correlation between the residual volume and the percentage of excessive weight loss was found mainly during the first postoperative year. Moreover, patients with a higher pre-surgical body mass index (BMI) (≥ 50 kg/m^2^) achieved less weight loss at 5 years, and BMI was one of the main factors of weight regain [17]. Accordingly, long-term LSG residual volume is an important parameter linked to success rate, as reported by Deguines et al. [18].

The restrictive actions elicited by LSG also provide changes in the mechanical response of gastric tissues. The reduction in capacity and near total fundectomy has direct consequences in terms of mechanical stimulation of specific gastric mechanoreceptors. The actions of these receptors in combination with hormonal effects and other forms of effector activation may play an important role in reaching satiety [19], although the mechanism of early satiety is still unclear [16]. For this reason, BS efficacy should be assessed on the basis of the elongation/deformation mechanical quantities related to food intake.

The aim of this work was to investigate how the basal volume at different intragastric pressures varies with inner diameter size. Furthermore, this study aimed to describe in a quantitative way the elongation strains registered by mechanoreceptors in different regions and layers of the sleeved stomach. The analysis of these surgical parameters was carried out by biomechanical computational tools and methods belonging to the bioengineering field. Computational finite-element models allow for the investigation of many different scenarios in a short time without performing laboratory tests. Moreover, many quantities, which would be impossible to measure in vivo or would require very invasive equipment, can be recorded.

In the future, computational models could be employed as support tools for decision-making and rational preoperative planning to maximize surgical effectiveness and reduce risks and complications.

## Materials and methods

**Fig. 1:**
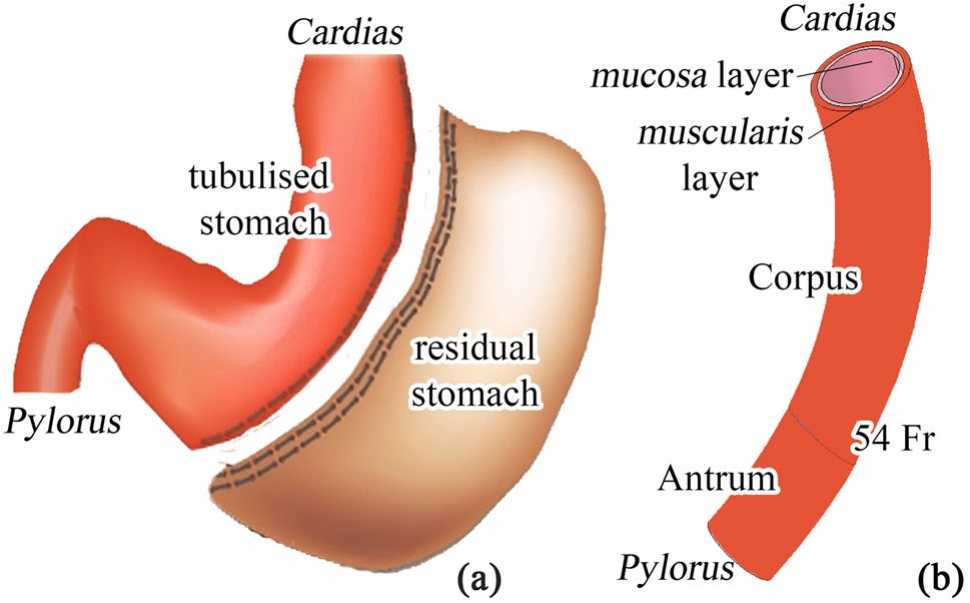
Sleeve gastrectomy

The computational investigation of stomach mechanics after LSG intervention requires the development of finite-element models of the sleeved structure. The action accounts for the geometrical characterization of gastric district by means of 3D CAD virtual solid modeling operations and finite-element discretization and the characterization of stomach tissue mechanics by means of constitutive formulations [20, 21]. Ten 3D CAD virtual solid models of stomachs treated by LSG were developed, simulating the use of different guide tubes to size the sleeved stomach (Fig. 1). The inner diameters varied from 27 to 54 Fr, while the length of the greater curvature was kept fixed at 150 mm.

The models included both the antrum and corpus regions because most of the fundus is usually removed during LSG [22], and separately described the inner mucosa–submucosa layer and the outer muscularis layer (Fig. 1b). Several measurements of gastric wall thickness were performed on excised stomach specimens during LSG using a digital feeler gauge (0–13 mm, 0.01 mm of resolution). The measurements mainly pertained to the corpus region and revealed an average thickness of 2.5 mm. An analysis of data from the literature on animal and human samples [23, 24] suggested considering that the muscularis layer is thicker than the mucosa. The estimated thicknesses for the mucosa and muscularis layers were 1 mm and 1.5 mm, respectively. Finite-element discretization was performed by means of 8-node hexahedral elements, whose size was set to 2 mm along the longitudinal and circumferential directions and to 0.5 mm along the thickness direction.

The constitutive analysis of stomach tissues aimed to describe the typical features of their mechanical behavior, such as fiber-reinforced configuration, nonlinear elasticity and time dependence, leading to an anisotropic visco-hyperelastic formulation. Model parameters were identified by analyzing data from mechanical experiments performed on swine samples at both the tissue and structure levels, as fully reported by Fontanella et al. [21]. Aiming to thoroughly investigate the mechanics of human tissues, the previous constitutive parameters were adapted by means of data from experiments performed on stomach samples obtained after LSG. This study did not include experiments on human and animal subjects, and consequently, no IRB approval or informed consent was required.

Subsequently, computational models were exploited to analyze the biomechanical functionality of sleeved stomachs. The model was kept fixed at both gastroesophageal and gastroduodenal junctions, and the inner cavity was inflated up to a pressure of 75 mmHg. The pressure of the lower esophageal sphincter ranges between 10 and 30 mmHg [25], and the intragastric pressure does not usually reach values higher than 30 mmHg. However, the assumed higher limit was chosen to investigate the mechanical response of gastric tissues in conditions of very high parietal stress. Computational assessments were performed by means of the general-purpose finite-element code Abaqus Standard 2018 (Dassault Systèmes, Simulia Corp., Providence, RI). All simulations were performed by means of a High-Performance Computing Server Fujitsu Primergy RX4770 equipped with two Intel Xeon E7 8890 v4 processors, 256 GB RAM and SSD HD. Each analysis required a mean execution time of 2 h when 20 threads were utilized.

## Results

**Fig. 2:**
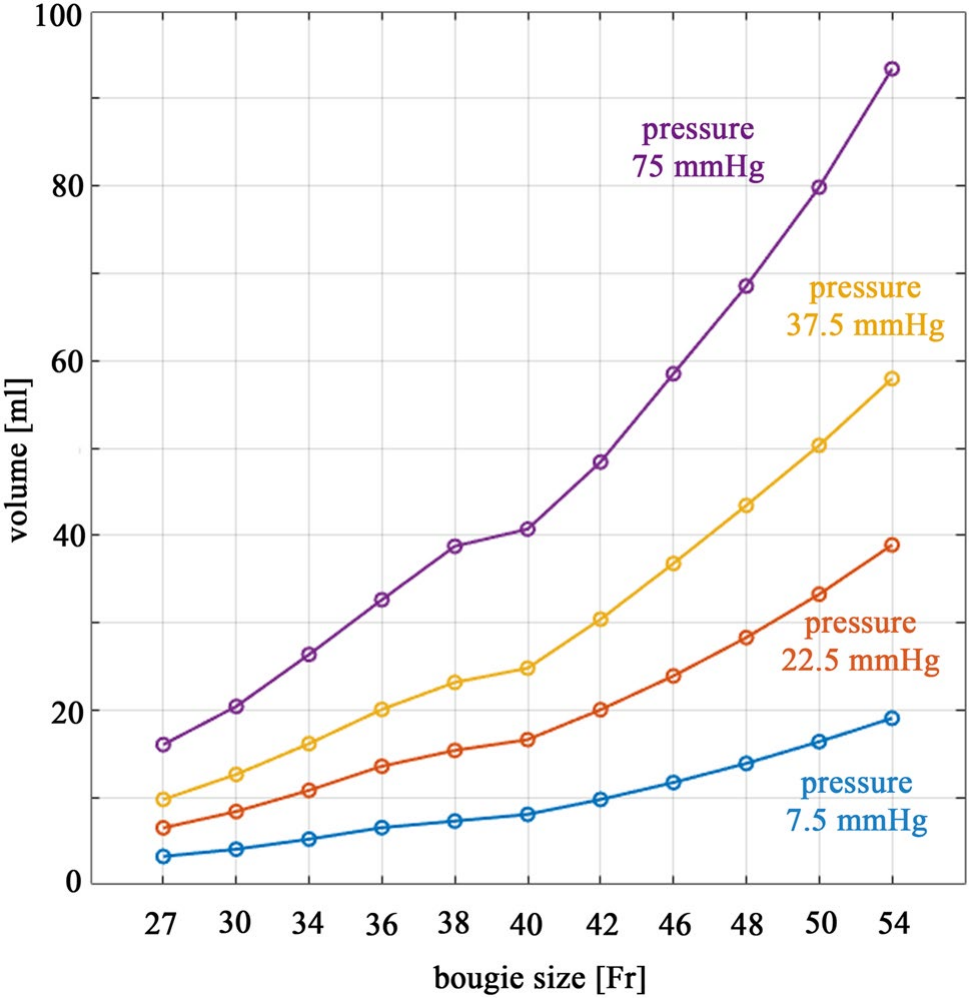
Volume vs bougie size

The in silico simulations provided values considered unmeasurable or difficult to measure in vivo, which allowed for the quantitative comparison of different LSG post-surgical configurations of the sleeved stomach. Model exploitation permitted rational detection of the basal volume at different intragastric pressure values. In Table 1, the basal volumes calculated at different inner pressures (7.5, 15, 22.5, 37.5 and 75 mmHg) are reported for different LSG bougie sizes, while in Fig. 2, the volume values corresponding to 7.5, 22.5, 37.5 and 75 mmHg of intragastric pressure are reported in a chart to better highlight the increasing trend. As an example, at an inner pressure of 75 mmHg, the basal volume of the 54 Fr configuration was approximately 5 and half times higher than that of the 27 Fr configuration (150.84 ml vs 27.30 ml).

**Table 1:** Volumes needed to reach different intragastric pressures, as 7.5, 15, 22.5, 37.5 and 75 mmHg, for each bougie size

Bougie size (Fr)	Basal volume at 7.5 mmHg (ml)	Basal volume at 15 mmHg (ml)	Basal volume at 22.5 mmHg (ml)	Basal volume at 37.5 mmHg (ml)	Basal volume at 75 mmHg (ml)
27	3.24	6.52	9.7	16.04	27.30
30	4.07	8.38	12.63	20.41	35.53
34	5.22	10.80	16.15	26.36	45.05
36	6.54	13.56	20.07	32.62	56.05
38	7.29	15.38	23.16	38.74	67.01
40	8.06	16.61	24.79	40.71	68.52
42	9.76	20.04	30.39	48.39	82.04
46	11.70	23.90	36.77	58.49	95.25
48	13.89	28.29	43.41	68.53	113.77
50	16.37	33.24	50.29	79.84	131.59
54	19.05	38.89	57.92	93.38	150.84

**Fig. 3:**
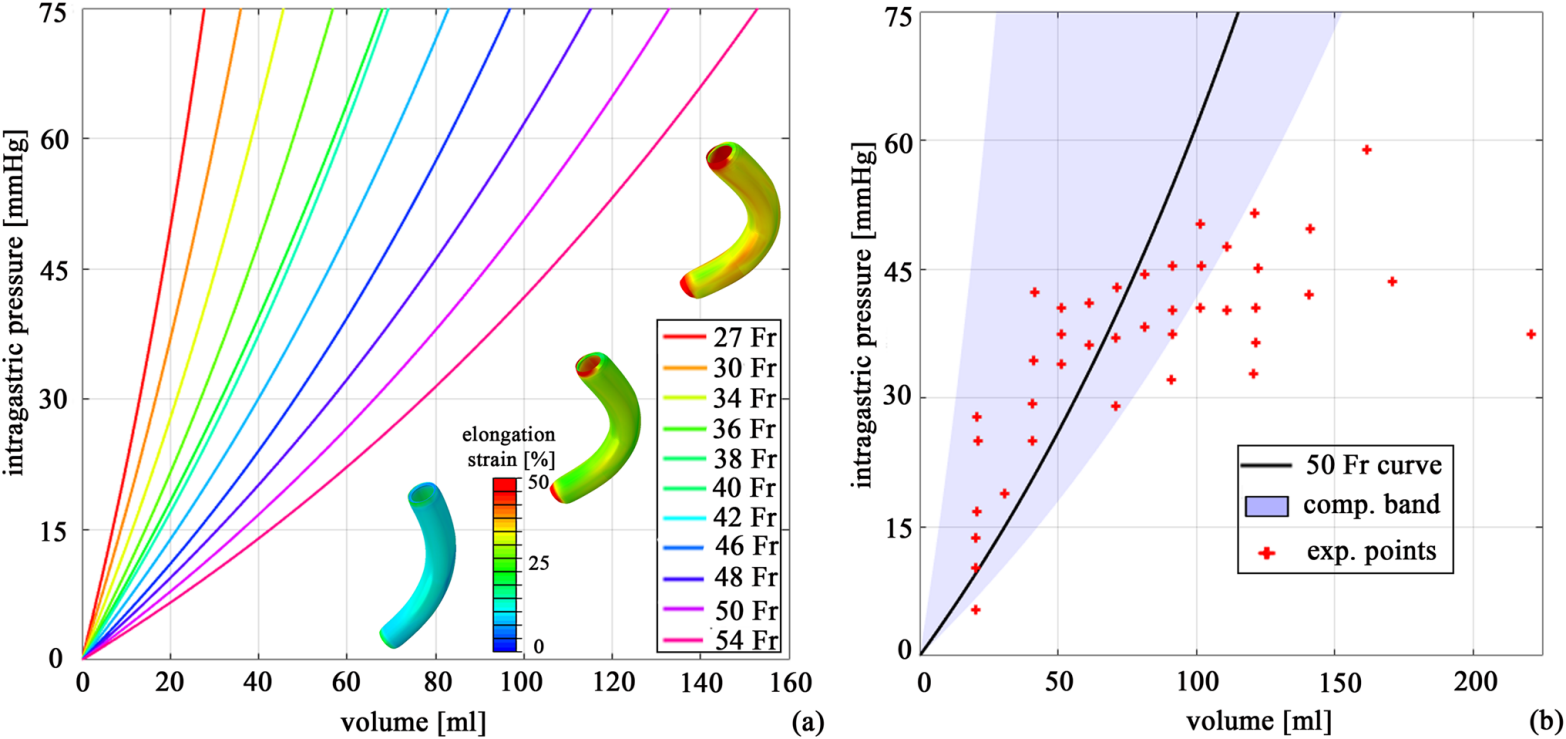
Pressure-volume behaviour

In Fig. 3a, the pressure–volume behavior is reported for each bougie size analyzed. Increasing the inner diameter size shifted the curves to the right: to reach the same intragastric pressure state, the inflated volume had to be higher when a larger bougie size was considered. The pressure–volume behavior showed an exponential trend, whose slope decreased as the bougie size increased. A comparison was performed between the computational band and the in vivo pressure–volume measurements after LSG, as reported in Yehoshua et al. [16]. The entire computational band was reported to highlight the effects of the sleeved stomach’s dimension. Most of the in vivo measurements (approximately 65%) fell in the computational band (Fig. 3b). The experimental points showed the high invariability among human sleeved stomachs obtained using a unique bougie size (50 Fr) due to inescapable geometrical differences, such as the length.

**Fig. 4:**
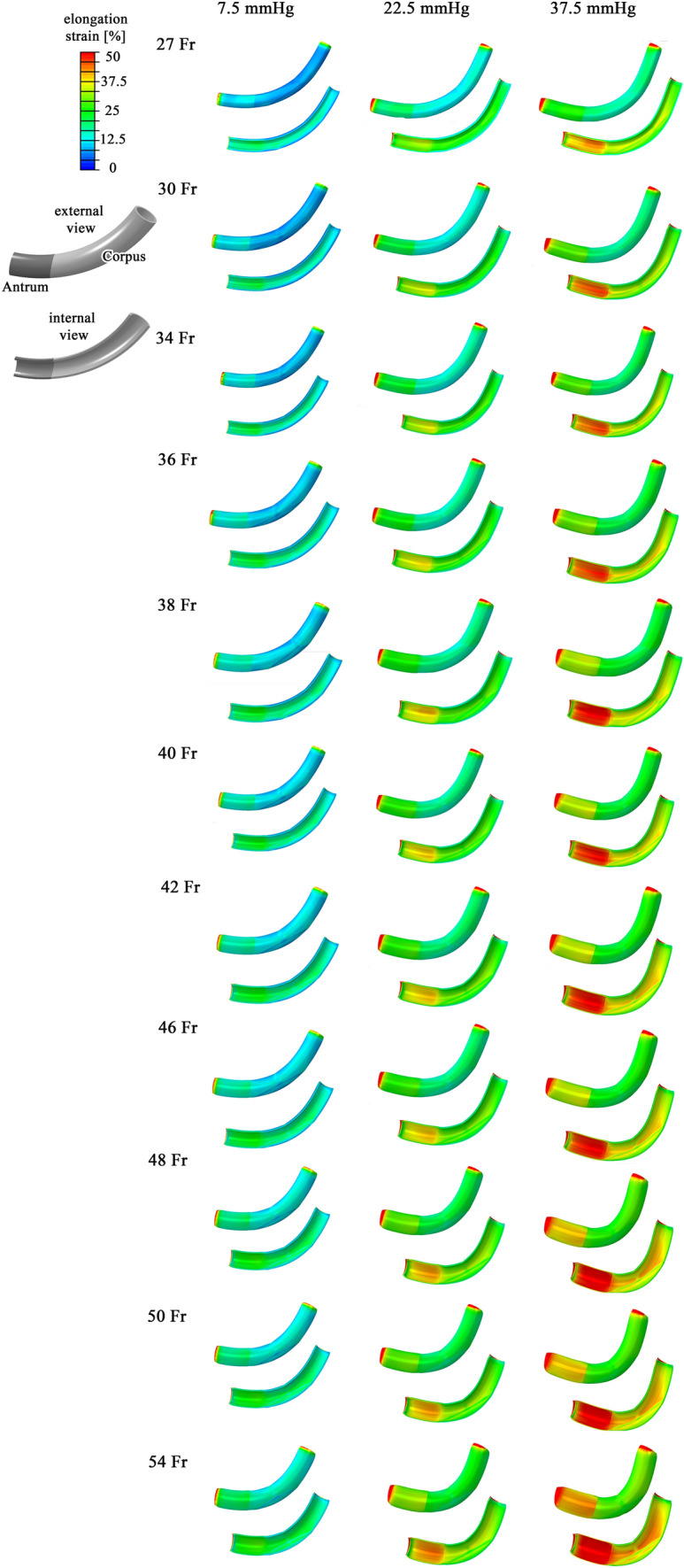
Computational results

**Fig. 5:**
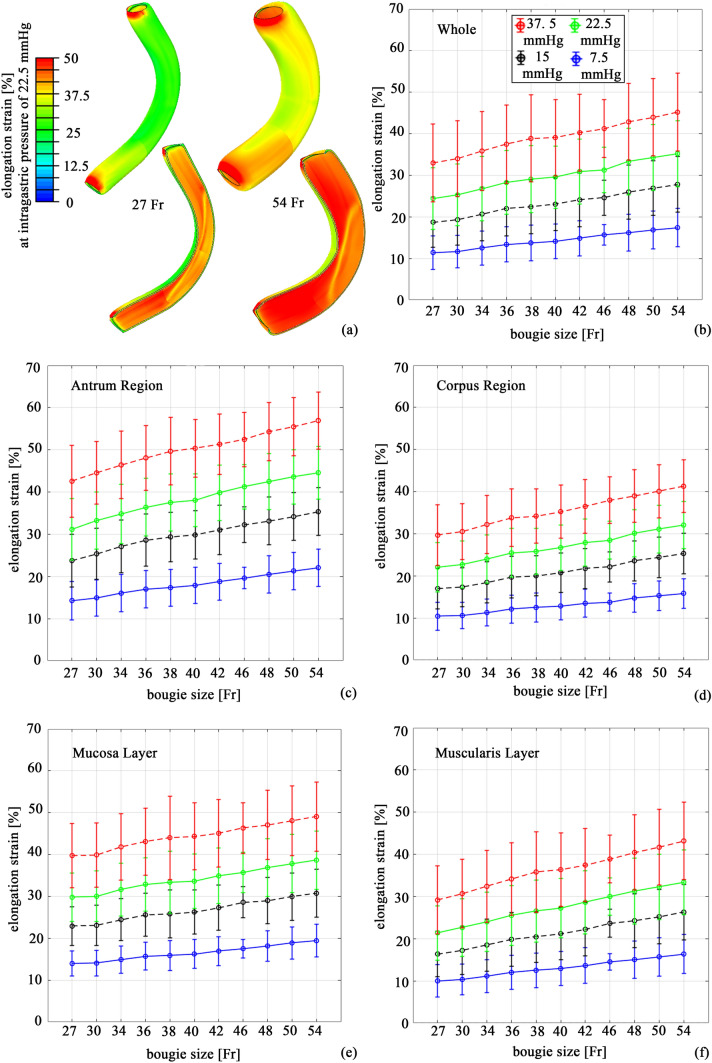
Elongation strain differentiated by stomach regions

In the scientific literature, the influence of stomach wall distension on the mechanisms of satiety is highly reported [26–28]. Computational analyses made it possible to quantitatively evaluate such mechanical stimulation. The distribution of elongation strain is reported in Fig. 4 for the different bougie sizes by means of contour plots. The results highlighted the greater stimulation of the mucosa layer than the muscularis layer. The data reported in Fig. 5 and Table 2 provide a thorough quantitative report of wall distension. Statistical distributions of computational results were provided for the whole model, different regions of the stomach, and different tissue layers.

**Table 2:** Elongation strains (mean [%] ± standard deviation [%]) computed for the whole model of the sleeve stomach and differentiated by region and by layer

	Whole model elongation strain [%]	Antrum region elongation strain [%]	Corpus region elongation strain [%]	Mucosa layer elongation strain [%]	Muscularis layer elongation strain [%]
27 Fr					
7.5 mmHg	11.42 (± 4.03)	14.30 (± 4.55)	10.43 (± 3.30)	13.96 (± 2.99)	10.00 (± 3.84)
15 mmHg	18.73 (± 6.00)	23.76 (± 6.25)	16.99 (± 4.82)	22.90 (± 4.56)	16.38 (± 5.42)
22.5 mmHg	24.41 (± 7.41)	31.15 (± 7.30)	22.09 (± 5.87)	29.80 (± 5.81)	21.39 (± 6.43)
37.5 mmHg	32.97 (± 9.42)	42.56 (± 8.48)	29.66 (± 7.20)	39.76 (± 7.65)	29.16 (± 8.08)
30 Fr					
7.5 mmHg	11.66 (± 3.90)	14.94 (± 4.29)	10.59 (± 3.09)	14.06 (± 3.06)	10.34 (± 3.69)
15 mmHg	19.31 (± 6.07)	25.36 (± 5.99)	17.33 (± 4.62)	23.08 (± 4.81)	17.22 (± 5.68)
22.5 mmHg	25.28 (± 7.43)	33.24 (± 6.73)	22.67 (± 5.55)	30.04 (± 6.06)	22.64 (± 6.77)
37.5 mmHg	33.99 (± 9.12)	44.54 (± 7.43)	30.53 (± 6.63)	39.92 (± 7.68)	30.69 (± 8.14)
34 Fr					
7.5 mmHg	12.52 (± 4.10)	16.03 (± 4.43)	11.31 (± 3.19)	14.91 (± 3.21)	11.49 (± 3.93)
15 mmHg	20.67 (± 6.44)	27.07 (± 6.42)	18.47 (± 4.83)	24.44 (± 5.02)	18.51 (± 6.16)
22.5 mmHg	26.79 (± 7.71)	34.85 (± 7.03)	24.01 (± 5.73)	31.65 (± 6.29)	24.00 (± 7.04)
37.5 mmHg	35.85 (± 9.49)	46.43 (± 7.96)	32.20 (± 6.88)	41.82 (± 7.94)	32.41 (± 8.56)
36 Fr					
7.5 mmHg	13.37 (± 4.19)	17.01 (± 4.41)	12.11 (± 3.28)	15.69 (± 3.32)	11.99 (± 4.05)
15 mmHg	21.99 (± 6.55)	28.60 (± 6.17)	19.70 (± 4.93)	25.59 (± 5.16)	19.85 (± 6.35)
22.5 mmHg	28.26 (± 7.70)	36.39 (± 6.81)	25.46 (± 5.76)	32.90 (± 6.37)	25.52 (± 7.08)
37.5 mmHg	37.47 (± 9.41)	48.18 (± 7.66)	33.81 (± 6.81)	43.09 (± 7.97)	34.15 (± 8.58)
38 Fr					
7.5 mmHg	13.75 (± 4.26)	17.35 (± 4.34)	12.51 (± 3.46)	15.88 (± 3.58)	12.49 (± 4.14)
15 mmHg	22.42 (± 6.50)	29.37 (± 5.88)	20.03 (± 4.75)	25.82 (± 5.70)	20.43 (± 6.10)
22.5 mmHg	29.08 (± 8.07)	37.52 (± 6.77)	25.81 (± 5.49)	33.39 (± 6.97)	26.55 (± 7.32)
37.5 mmHg	38.85 (± 10.47)	49.66 (± 8.01)	34.20 (± 6.39)	44.03 (± 9.95)	35.82 (± 9.54)
40 Fr					
7.5 mmHg	14.13 (± 4.14)	17.88 (± 4.26)	12.86 (± 3.23)	16.25 (± 3.42)	12.92 (± 4.03)
15 mmHg	23.04 (± 6.34)	29.83 (± 5.74)	20.74 (± 4.67)	26.28 (± 5.26)	21.20 (± 6.17)
22.5 mmHg	29.57 (± 7.50)	38.06 (± 6.30)	26.69 (± 5.40)	33.63 (± 6.45)	27.25 (± 7.06)
37.5 mmHg	39.08 (± 9.18)	50.36 (± 6.88)	35.25 (± 6.25)	44.36 (± 7.97)	36.37 (± 8.72)
42 Fr					
7.5 mmHg	14.84 (± 4.27)	18.78 (± 4.35)	13.50 (± 3.31)	16.93 (± 3.48)	13.63 (± 4.21)
15 mmHg	24.09 (± 6.46)	31.00 (± 5.83)	21.75 (± 4.77)	27.31 (± 5.37)	22.23 (± 6.31)
22.5 mmHg	30.92 (± 7.80)	39.84 (± 6.55)	27.90 (± 5.56)	34.90 (± 6.62)	28.63 (± 7.51)
37.5 mmHg	40.24 (± 9.23)	51.33 (± 7.14)	36.48 (± 6.41)	45.08 (± 8.08)	37.47 (± 8.69)
46 Fr					
7.5 mmHg	15.66 (± 2.51)	19.62 (± 2.55)	13.75 (± 2.14)	17.52 (± 2.23)	14.52 (± 1.99)
15 mmHg	24.59 (± 4.24)	32.24 (± 4.19)	22.14 (± 3.53)	28.61 (± 3.75)	23.65 (± 3.34)
22.5 mmHg	31.25 (± 5.46)	4.12 (± 5.25)	28.46 (± 4.46)	35.69 (± 4.79)	30.01 (± 4.39)
37.5 mmHg	41.23 (± 6.92)	52.46 (± 6.47)	37.98 (± 5.57)	46.32 (± 6.06)	38.88 (± 5.61)
48 Fr					
7.5 mmHg	16.18 (± 4.44)	20.47 (± 4.38)	14.73 (± 3.39)	18.16 (± 3.68)	15.05 (± 4.44)
15 mmHg	25.99 (± 6.49)	33.13 (± 5.65)	23.57 (± 4.73)	29.00 (± 5.55)	24.27 (± 6.36)
22.5 mmHg	33.31 (± 7.96)	42.51 (± 6.66)	30.13 (± 5.57)	36.87 (± 6.84)	31.28 (± 7.84)
37.5 mmHg	42.85 (± 9.24)	54.27 (± 6.88)	38.98 (± 6.25)	47.08 (± 8.21)	40.43 (± 8.92)
50 Fr					
7.5 mmHg	16.85 (± 4.57)	21.32 (± 4.42)	15.33 (± 3.49)	18.88 (± 3.83)	15.71 (± 4.55)
15 mmHg	26.90 (± 6.59)	34.17 (± 5.66)	24.44 (± 4.82)	29.93 (± 5.64)	25.22 (± 6.47)
22.5 mmHg	34.28 (± 7.96)	43.59 (± 6.40)	31.10 (± 5.62)	37.83 (± 6.97)	32.27 (± 7.79)
37.5 mmHg	43.97 (± 9.28)	55.45 (± 6.89)	40.07 (± 6.28)	48.10 (± 8.30)	41.67 (± 9.00)
54 Fr					
7.5 mmHg	17.42 (± 4.62)	22.08 (± 4.41)	15.86 (± 3.51)	19.42 (± 3.89)	16.37 (± 4.62)
15 mmHg	27.82 (± 6.66)	35.34 (± 5.65)	25.31 (± 4.83)	30.74 (± 5.72)	26.29 (± 6.60)
22.5 mmHg	35.19 (± 7.88)	44.58 (± 6.24)	32.06 (± 5.54)	38.66 (± 6.97)	33.38 (± 7.72)
37.5 mmHg	45.19 (± 9.32)	56.94 (± 6.80)	41.28 (± 6.25)	49.63 (± 8.31)	43.18 (± 9.18)
Mean					
7.5 mmHg	14.34 (± 4.09)	18.16 (± 4.22)	13.00 (± 3.22)	16.51 (± 3.33)	13.11 (± 3.95)
15 mmHg	23.23 (± 6.21)	29.99 (± 5.75)	20.95 (± 4.66)	26.70 (± 5.14)	21.39 (± 5.90)
22.5 mmHg	29.85 (± 7.54)	38.45 (± 6.55)	26.95 (± 5.51)	34.12 (± 6.42)	27.54 (± 7.00)
37.5 mmHg	39.24 (± 9.19)	50.19 (± 7.33)	35.49 (± 6.45)	44.42 (± 8.01)	36.38 (± 8.46)

The values were calculated at different intragastric pressure conditions, as 7.5 mmHg, 15 mmHg, 22.5 mmHg and 37.5 mmHg

The elongation strain increased when the bougie size increased for all regions and layers, reaching maximum values in the 54 Fr configuration, even though the intragastric pressure state was the same. The models with higher bougie sizes displayed greater mechanical stress, reaching a greater elongation strain distribution, which was easily detectable thanks to colormaps (Fig. 4). These results were easily explicable according to Laplace’s law [29]: the tension of the wall is proportional to both the inner pressure and radius of the tube. Higher bougie sizes corresponded to a higher radius, and thus, to a more elongated gastric wall.

The regions and layers that recorded the highest elongation strain values were the antrum and mucosa layer, respectively. If all the bougie sizes were considered, the mean elongation strain of the antrum was approximately 11% higher than that of the corpus at an intragastric pressure of 22.5 mmHg and approximately 25% higher than that of the corpus at 37.5 mmHg. Considering the mucosa and muscularis layers, the elongation strain of the mucosa layer was 3% and 7% higher than that of the muscularis layer at intragastric pressures of 7.5 and 22.5 mmHg, respectively, reaching a difference of 8% when a pressure of 37.5 mmHg was considered.

## Discussion

LSG acts directly by reducing food and caloric intake and modifying total stomach capacity and parietal distensibility and indirectly impacting meal-induced satiety via brain–gut neuro-hormonal loops [30, 31]. Food ingestion induces mechanical stimulation of the gastric wall and promotes the release of satiety signals [32–35]. In the past, most investigations have focused on the assessment of qualitative, not quantitative, activation of gastric mechanoreceptors. Therefore, clinical investigations and experimental methods allow for a partial analysis of the influence of bariatric surgery on stomach functionality. Specifically, the experimental evaluation of stomach capacity and stiffness is feasible, but the measurement of mechanical stimulation on tissues and receptors, such as the stress and strain fields, is challenging. Furthermore, experimental investigations are expensive and time consuming, as they require extensive experimental sampling that may make data processing extremely difficult, and interspecimen variability can be an unavoidable limitation. On the other hand, experimental activities can be exploited to define, identify and validate computational models of biological structures [20, 21, 23, 36]. As a consequence, computational methods allow for the expansion of experimental results to widened scenarios, taking into consideration many different configurations of biological factors, as well as many different surgical situations and procedures, thus, providing information that experimental activities may not be able to provide, such as the stress and the strain fields involved in the stimulation of gastric mechanoreceptors.

LSG is one of the most performed operations in the bariatric operation. Although it is considered safe and effective, this approach is not flawless and without complications. In fact, LSG is sometimes linked to a worsening of GERD or de novo GERD [11, 13], due to stricture, twist/torsion and retained fundus, with or without herniation [37]. Moreover, optimal sleeve construction is still a controversial issue [15]; hence, bioengineering methods are needed for further investigations. For this reason, this work focused on the LSG procedure, pointing out quantitative characterization of regions and more stressed wall layers. Due to the highly versatile nature of computational models, many scenarios can be assessed. In this paper, ten different bougie sizes were analyzed, and computational results were employed to discriminate modifications among the different models in a rational and quantitative manner. The pressure–volume behavior and the elongation strain distribution were calculated. The first was assessed at the end of the surgical procedure [16], but only few patients were enrolled and LSG was performed using a single type of orogastric bougie (50 Fr). Even though these measurements were related to a small patients group, it was a necessary preliminary step to assess the reliability and the relevance of the computational results proposed in this paper. However, to define the main differences among surgical LSG techniques, further data taking into consideration different bougie sizes and more prolong in vivo pressure assessments are necessary. Such in vivo experimentations can be fairly replaced by simulations of a validated computational model, thus, reducing complexity and costs dramatically. For this reason, a stronger multidisciplinary collaboration between clinicians and bioengineers should be advocated to exploit all the potentials of computational models coupled with available clinical data.

In addition, the computational approach can be extended to indagate other well-established bariatric procedures or new and innovative methods, as reported in other studies [21, 38]. The main limitation includes the use of a simplified geometry (hollow cylinders), and digestion and absorption phenomena, effect of gastric motility and interaction between the food bolus and gastric wall were not considered yet. Moreover, the influence of the distance from the pylorus, the EG insertion angle, the mechanisms promoting the occurrence of hiatal hernia, and how these factors may influence plasma levels of satiety-related hormones were not investigated. As an example, fluid structure interaction analyses are under development, aiming to analyze the influence of the post-surgical His angle on the development of GERD.

Furthermore, future studies will match computational geometries with patient imaging data, and the suture lines, considering both staples and stitches, will be included in the computational models. Thus, it will be possible to assess the elongation and stress distribution along the staple lines to predict any eventual points of weakness and prevent surgical complications. For these reasons, in the future, the surgical procedure should be customized according to patient’s medical needs integrated by computational models. The customization will consist of combing patient basal data (BMI, pre-surgical stomach capacity and morphology, His’s angle degrees, presence of reflux events…) with pre-surgical stomach morphology (i.e., by means of MRI scan) to tailor the best post-surgical stomach configuration that allows to achieve the best results in terms of weight loss avoiding negative surgical outcomes.

## Conclusion

The proposed work highlighted the potential of bioengineering methods to perform quantitative assessments and investigations of BS. Specifically, the computational modeling approach allowed for the easy evaluation of the influence of LSG parameters on the basal volume of the sleeved stomach at different intragastric pressures. Furthermore, the computation granted the possibility to rationally quantify elongation strains as a measure of gastric wall distension within the sleeved stomach wall. The analyses established the region and layer of the sleeved stomach that recorded the greater elongation strains, which were the antrum and the mucosa layer, respectively. Such information is necessary to evaluate the stimulation of the gastric mechanoreceptors primarily involved in the mechanisms of satiety and satiation. Higher values of elongation strains were recorded in models with larger bougie sizes. Therefore, the optimal bougie size should be a compromise between a significant elongation of the gastric wall and a fair reduction of stomach size on the basis of preoperative patient characteristics.

Computational models could be a powerful tool to improve the results of LSG and properly address the main drawbacks of BS.
